# Mittheilungen Uber Das Badische Veterinarwesen

**Published:** 1882-04

**Authors:** 


Mittheilungen uber das badische Veterinarwesen, in der Jahren 1874 bis 1880 bearbeitel von Medizinalrath A. Lydtin, mit 12 lithographirten Tafeln. Karlsruhe: 1882.

Report upon the veterinary police regulations for the years 1874 to 1880, by the medical superintendent, A. Lydtin. With twelve lithographic plates.

The veterinarian of this country may well view with envy the advanced condition of his art in Germany, where such a report as the one before us is published by the government.

We learn from it how carefully regulated is the practice of veterinary medicine, and how perfect are the sanitary provisions for the care of the lower animals.

The book is a large one. It contains chapters describing the organization of the veterinary police, the condition and work of the veterinarians, with statistics, tables and charts showing the prevalent diseases.

It furnishes the model which we hope our States will eventually imitate.

